# Perceived humiliation during admission to a psychiatric emergency service and its relation to socio-demography and psychopathology

**DOI:** 10.1186/1471-244X-13-217

**Published:** 2013-08-29

**Authors:** Marit F Svindseth, Jim A Nøttestad, Alv A Dahl

**Affiliations:** 1 Aalesund University College, P.O. Box 1517, N-6025 Aalesund, Norway; 2 Department of Forensic Psychiatry, Brøset, St. Olav’s Hospital, 7440 Trondheim, Norway; 3 Department of Oncology, Oslo University Hospital, Radiumhospitalet, 0424 Oslo, Norway; 4 University of Oslo, 0316 Oslo, Norway

**Keywords:** Humiliation, Psychiatry, Narcissism, Psychopathology, Violence

## Abstract

**Background:**

There is a lack of empirical studies of patients’ level of humiliation during the hospital admission process and its implications for the clinical setting. We wanted to explore associations between self-rated humiliation and socio-demography and psychopathology in relation to admission to a psychiatric emergency unit.

**Methods:**

Consecutively admitted patients (N = 186) were interviewed with several validated instruments. The patients self-rated humiliation by The Cantril Ladder, and 35% of the sample was defined as the high humiliation group.

**Results:**

Final multivariate analysis found significant associations between compulsory admission, not being in paid work, high scores on hostility, and on entitlement, and high levels of humiliation. No significant interactions were observed between these variables, and the narcissism score was not a confounder concerning humiliation.

**Conclusions:**

High level of humiliation during the admission process was mainly related to patient factors, but also to compulsory admission which should be avoided as much as possible protecting the self-esteem of the patients.

## Background

According to Lazare & Levy [1]: “Humiliation is the emotional response of people to their perception that another person or group has *unfairly or unjustly* lowered, debased, degraded, or brought them down to an inferior position, that they are not receiving the respect and dignity they believe they deserve”. While this definition focused on the experience of unfair or unjust treatment by others, Torres and Bergner [2] emphasized the *loss of the status to claim a status* as a central element of humiliation: “When a humiliation annuls the very standing of individuals as eligible to make status claims on their own behalf, these individuals have been nullified as participating actors in the relational domain, or community in which the humiliation has taken place”.

According to both perspectives having a mental disorder is a potent risk factor for humiliation if the symptoms are displayed in front of others. To be forced into treatment for such disorders through compulsory admission, increases the risk still further. In line with Lazare & Levy [1] most individuals experience such an admission as unjust, and according to the formulation of Torres & Bergner [2] such an admission, irrespective of its perceived fairness, constitutes a loss of status in relation to the norm of social status claims that most individuals are eligible to make. In spite of this, stigma and shame rather than humiliation have been studied in patients with mental disorders.

Birchwood et al. and Rooske & Birchwood [3,4] found that humiliation was strongly associated with compulsory admission in patients with schizophrenia, and particularly so in those with co-morbid depression. Based on interviews, Svindseth et al. [5] explored the perception of humiliation related to the admission process in a sample of 102 patients hospitalized at an emergency unit in Norway. They found significant associations between humiliation and the patients’ feeling that the admission “was not right” and use of physical force during admission [5]. Later Svindseth et al. [6] reported that the level of perceived humiliation was significantly reduced during the admission in the “more improved” but not in the “less improved” patients. In the present study our group further investigate perceived humiliation in the same patient sample addressing two research questions: 1) Are there significant associations between perceived humiliation and socio-demographic and psychopathological variables? 2) Which of these variables are most strongly associated with perceived humiliation?

## Methods

### Setting

Aalesund Hospital is located in the city of Aalesund at the North-Western coast of Norway. The emergency unit has four wards: two closed ones (8 beds each) and two open ones (8 beds and 10 beds, respectively), all with separate patient rooms. The hospital serves a geographical sector of about 95,000 inhabitants ≥ 18 years of age.

### Patient sampling

Admitted patients to the two closed acute wards from March 1, 2005 to October 15, 2006, were consecutively invited to the study if they were eligible. Exclusion criteria were dementia or organically based confusion, manic or hypomanic states, re-admittance during the sampling period, poor ability to speak Norwegian, or discharge within 48 hours. Both involuntary and voluntary admitted patients were eligible. All involuntary patients were invited to the study, but due to a majority of voluntary patients, only those admitted on specifically defined days of the week were invited. All patients had an interview within three days after admission, except a minority who were interviewed within the first week due to the severity of their mental state at admission. During the sampling period 191 patients with involuntary status were admitted, and 78 did not meet the eligibility criteria, 8 declined to take part or withdrew their consent, and 7 were lost due to administrative reason. This left 98 involuntary patients for the study. On the defined days, 160 voluntary patients were admitted, 48 did not meet the eligibility criteria, 13 declined to take part or withdrew their consent, and 11 were lost due to administrative reason. This left 88 voluntary patients for the study. The total sample thus consisted of 186 patients.

### Measurements

#### Interview-based instruments

*The Brief Psychiatric Rating Scale (BPRS)* is a clinician-rated test designed to assess status of and changes in severity of psychopathology [7,8] with focus on symptoms of psychosis. We used the 24 items version, and the time frame of evaluation was the day of the interview. Items were rated on a 7-point Likert-like scale anchored from 1 (not present) to 7 (extremely severe), and thus higher scores represent more psychopathology. We used a version of BPRS with explanations of each of the rating points. We reported on BPRS total score as well as the following subscales: Thinking disturbance (Cronbach’s alpha 0.61); Hostility/suspiciousness (alpha 0.61) Anxiety/depression (alpha 0.57) and Activation (alpha 0.61).

Eight experienced registered psychiatric nurses, trained by the first author, did the patient interviews and assisted the patients in filling in the self-report forms if necessary. Training of the nurses involved study of written material on the BPRS, taking part in group-discussions and making three patient interviews supervised by the first author. Reliability testing of the eight interviewers showed correlation coefficients of 0.87 – 0.97 compared to those of the supervisor and between the interviewers of 0.74- 0.97 based on the BPRS scorings of three patients. *Suicidality* was evaluated on admission by a psychiatrist and the rating was dichotomized as suicidal/not-suicidal.

*The Scale for Prediction of Aggression and Dangerousness* has been modified in Norwegian studies [9]. Violence was recorded from the first professional or police contact leading to admission to discharge through both observations in the wards and documentation in the medical records. Violence was classified according to the Intensity subscale into: “No violence” “Threats”, “Mild violence”, “Moderate violence” and “Severe violence”, In the logistic regression analyses these scorings were recoded into two categories: *Mild violence* = “no violence”, “threats” and “mild violence” and *severe violence* = “moderate” and “severe” violence.

*ICD-10 diagnoses*[10] are mandatory in Norway and were given by the patient-responsible psychiatrist at the end of the index admission. Only the main diagnosis was used in this study.

*Global Assessment of Functioning (GAF)* is an observer-based rating scale for the current overall functioning of a patient on a continuum from the most severe mental disorder to complete mental health that was defined as Axis V of the DSM-IV. Scale values range from 1 (sickest individual) to 100 (the healthiest individual). A Norwegian study examined the reliability of the GAF split into GAF Functions (GAF-F) and GAF Symptoms (GAF-S) [11]. Both GAF-F and GAF-S were found to be highly reliable and had a correlation of rho = 0.61. In our study, the psychiatrists scored the GAF-F and the GAF-S at the admission interview [11].

*Socio-demographic variables.* Level of basic education was divided into two classes (≤12, >12 years) based on completed school years; work status was dichotomized (paid work or self-employed, versus unemployed or pensioned). Civil status was divided into paired (married, cohabiting) and non-paired relationships.

### Patient-rated instruments

*Perceived humiliation* was measured with the Cantril Ladder Measure, which is a visual, analogue scale from 1 (minimum humiliation) to 10 (maximum humiliation). The ladder is considered a general scale with good psychometric properties [12,13], and has been widely used in studies of patient experiences. The interviewer asked for perceived humiliation during the admission process, and read an instruction to the patient before he/she scored the Ladder, explaining that they should score the level of perceived emotional degradation and feeling of being of less worth. They were also given an explanation of the two endpoints of the Ladder. We dichotomized the total sample scores as close as possible to the 67-percentile implicating a cut-off score of ≥5 on the Ladder, and thereby 65 patients (35%) belonged to the high humiliation group and 121 (65%) to the low humiliated group.

*The Narcissistic Personality Inventory 29 item version (NPI).* We used the NPI-29 developed by Kansi [14] and further validated by Svindseth et al. [15]. The NPI-29 consists of 29 dual statements among which one is considered indicative of narcissism. Each statement is scored ‘yes’ or ‘no’, and there is no time limit as to the evaluation. Based on summation of the relevant items, the total NPI-29 score was calculated. Internal consistency values for the NPI-29 at admission were for the NPI-29 total Cronbach’s alpha = 0.85, and for the subscales: Leadership/Power (Factor 1) 0.66, Exhibitionism/Self-admiration (Factor 2) 0.72, Superiority/Arrogance (Factor 3) 0.57 and Uniqueness/Entitlement (Factor 4) 0.61. *The Hospital Anxiety and Depression Scale (HADS)* is a self-rating scale consisting of seven items measuring anxiety (HADS-A) and seven items measuring depression (HADS-D) during the last week [16]. The HADS-D focuses mainly on reduced ability to feel pleasure (anhedonia), and the HADS-A on generalized anxiety relating to worries and fear of what might happen in the future. Each item has scores from 0 (minimum presence) to 3 (maximum presence). The internal consistencies of the HADS-D and the HADS-A on admission were alpha = 0.85 and 0.82, respectively.

### Statistical analyses

Continuous measures were analyzed by paired sample t-tests, and categorical variables were examined with the *χ*^2^ test. Skewed distributions were examined with non-parametric tests as appropriate. Internal consistencies of scales were examined with Cronbach’s coefficient alpha. Statistically significant group differences were examined for clinical significance by means of effect sizes (ESs), and for continuous variables we used Cohen’s coefficient d and for 2 × 2 contingency tables the differences between arcsine transformed proportions [17,18]. ES values ≥0.40 were considered as clinically significant based on the recommendations of Cohen [19].

The strength of associations between independent variables and *humiliation* (dichotomized as high or low) was examined with univariate and multivariate logistic regression analysis. For both the BPRS and the NPI-29 the subscale scores correlated rho ≥ 0.65 with the total scores, so only the subscale scores were entered in the multivariate analyses. The GAF symptom and function scores showed rho = 0.77, and only function was entered in the multivariate analysis, since the BPRS covered symptoms. Since the high humiliation group had N = 65, we could only enter six variables into the multivariate analysis, and we therefore tested several models for relevant variables to be entered. The data were analyzed on SPSS (PASW) for PC version 18.0. The significance level was set at p < 0.05, and all tests were two-tailed.

### Ethics

The Regional Committee of Ethics in Medical Research of Mid-Norway, and The Norwegian Data Inspectorate approved the study. All patients gave written informed consent after oral and written information.

## Results

### Findings concerning socio-demography and humiliation

The high humiliation group contained a statistically significant higher proportion of patients with lower education and not being in paid work compared to the low humiliation group (Table 1). Compulsory admission, a diagnosis of schizophrenia and longer duration of the admission were statistical significantly more common in the high versus low humiliation group. The differences in work status and admission status reached clinical significance.

**Table 1:** Characteristics of high (N = 65) and low humiliation (N = 121) groups and the total sample (N = 186)

**Variables**	**High humiliation**	**Low humiliation**	**p-value**	Effect size*	**Total sample**
	**(N = 65)**	**(N = 121)**			**(N = 186)**
Age at admission, mean (SD)	38.1 (14.1)	37.0 (13.0)	0.59		37.3 (13.4)
*Sex, N (%)*			0.89		
Males	38 (59)	72 (60)			110 (59)
Females	27 (41)	49 (40)			76 (41)
*Relationship status, N (%)*			0.87		
Paired	17 (26)	33 (27)			50 (27)
Non-paired	48 (74)	88 (73)			136 (73)
*Level of education, N (%)*			0.04	0.33	
≤ 12 years	60 (92)	98 (81)			158 (85)
> 12 years	5 (8)	23 (19)			28 (15)
*Work status, N (%)*			0.003	0.48	
In paid work	9 (14)	41 (34)			50 (27)
Not in paid work	56 (86)	80 (66)			136 (73)
*Admission status, N (%)*			<0.001	0.95	
Compulsive admission	53 (82)	45 (37)			98 (53)
Voluntary admission	12 (18)	76 (63)			88 (47)
*Diagnosis at discharge, N (%)*			<0.001		
Non-psychotic disorders	23 (35)	61 (50)			84 (45
Mood disorders	14 (22)	40 (33)			54 (29)
Schizophrenia	28 (43)	20 (17)			48 (26)
*No of previous admissions, N (%)*			0.09		
None	29 (45)	70 (58)			99 (53)
One or more	36 (55)	51 (42)			87 (47)
*Duration of this admission, N (%)*			<0.001		
2 weeks or less	23 (35)	78 (65)			101 (54)
3 – 4 weeks	22 (34)	28 (23)			50 (27)
5 – 28 weeks	20 (31)	15 (12)			35 (19)

*Effect size is only given if p-value is significant and cannot be calculated for three-ways chi-square tests.

### Findings concerning psychopathology and humiliation

The high humiliation group scored statistically significant lower on the GAF-Function and GAF-Symptom scales compared to the low humiliation group (Table 2). The high humiliation group was also statistically significant less suicidal but more violent than the other group. The BPRS total score as well as all the subscale scores were statistically significant higher in the in the high versus the low humiliation group, and the same significance pattern was observed for the NPI-29 scores.

**Table 2:** Psychopathology of high (N = 65) and low humiliation (N = 121) groups and the total sample (N = 186)

**Variables**	**High humiliation**	**Low humiliation**	**p-value**	Effect size*	**Total sample**
	**(N = 65)**	**(N = 121)**			**(N = 186)**
*Admission GAF-scores, mean (SD)*					
GAF-Function	35.8 (11.8)	41.4 (10.3)	0.003	0.52	41.4 (10.7)
GAF-Symptoms	38.1 (11.2)	43.1 (10.1)	0.003	0.48	39.5 (11.1)
*Sucidal at admission, N (%)*	20 (31)	68 (56)	0.001	0.51	88 (47)
*Violence, N (%)*			0.001	0.52	
Mild	24 (37)	76 (63)			100 (54)
Severe	41 (63)	45 (37)			86 (46)
*BPRS scores, mean (SD)*					
Total	61.3 (16.1)	51.7 (12.4)	<0.001	0.70	55.1 (14.5)
Thinking disturbance	8.6 (4.1)	5.8 (3.4)	<0.001	0.77	6.8 (3.9)
Anxiety/depression	8.8 (4.3)	10.4 (3.7)	0.01	0.41	9.8 (4.0)
Hostility/suspicipouness	8.7 (3.8)	5.4 (3.2)	<0.001	0.96	6.6 (3.7)
Activation#	8.0 (4.2)	6.5 (3.3)	0.03	0.41	7.0 (3.7)
*HADS scores, mean (SD)*					
Depression#	8.9 (4.8)	9.1 (4.6)	0.95		9.0 (4.7)
Anxiety	12.4 (5.1)	11.8 (5.0)	0.29		12.0 (5.0)
*NPI-29 scores, mean (SD)*					
Total#	9.7 (6.2)	6.4 (4.2)	<0.001	0.66	7.5 (5.2)
Leadership/power (factor 1)#	2.8 (2.0)	2.0 (1.8)	0.003	0.43	2.3 (1.9)
Exhibitionism (factor 2)#	2.1 (2.0)	1.3 (1.5)	0.023	0.47	1.6 (1.8)
Superiority/Arrogance (factors 3)#	2.1 (1.7)	1.4 (1.2)	0.009	0.50	1.6 (1.4)
Entitlement/Uniqueness (factor 4)#	2.7 (2.0)	1.8 (1.6)	0.001	0.51	2.1. (1.8

# Indicate the use if non-parametric statistical tests due to skewed distributions. *Effect size is only given if p-value is significant and cannot be calculated for three-ways chi-square tests.

#### Strengths of association between independent variables and high humiliation

The univariate analyses confirmed the descriptive findings (Table 3). When we adjusted for the level of narcissism using the NPI-29 total score, none of the significant associations were modified. We made multivariate analyses of the independent variables in four groups, in order to identify the most potent ones to include among the six variables that could be entered in the final multivariate analysis. The variables “violence” and BPRS hostility/suspiciousness correlated strongly (rho = 0.60). We therefore decided to use BPRS hostility/suspiciousness in the multivariate analyses together with work status, GAF-Function, compulsory admission, NPI-29 Superiority, and NPI-29 Entitlement. In the final multivariate analysis, not being in paid work, compulsory admission, BPRS Hostility/Suspiciousness, and NPI-29 Entitlement were significantly associated with high humiliation. No significant interactions were observed between these variables.

**Table 3:** Bivarate and multivariate logistic regression analyses with high humiliation as dependent variable (low humiliation = reference)

**Independent variables**	**Univariate**			Group multivariate*		
	**OR**	**95% CI**	**p**	**OR**	**95% CI**	**p**
*Level of education*^*a*^				2.43	0.86 – 6.88	0.094
≥12 years (reference)	1.00					
<12 years	2.81	1.02 – 7.80	0.046			
***Working status***^***a***^				**2.96**	**1.30 – 6.62**	**0.008#**
In paid work (reference)	1.00					
Not in paid work	3.19	1.44 – 7.08	0.003			
***Compulsory admission***^***b***^	7.46	3.61 – 15.43	<0.001	**5.63**	**2.54 – 12.53**	**<0.001**
*Diagnosis at discharge*^*b*^						
Non-psychotic (reference)	1.00					
Mood disorder	0.93	0.43 – 2.02	0.85	1.22	0.50 – 2.96	0.660
Schizophrenia	3.71	1.76 – 7.84	0.001	2.06	0.90 – 4.74	0.089
*Duration of admission*^*b*^						
≤2 weeks (reference)	1.00					
3 – 4 weeks	2.27	1.29 – 5.51	0.008	2.04	0.91 – 4.54	0.082
5 – 28 weeks	4.52	2.00 – 10.22	<0.001	3.20	1.29 -7.94	0.012
*GAF-scores*						
**GAF-Function**	**0.96**	**0.93 – 0.98**	**0.003**			
GAF-Symptoms	0.95	0.93 – 0.98	0.001			
*Violence§*						
Mild (reference)	1.00					
Severe	2.89	1.55 – 5.39	0.001			
*BPRS subscales*						
Total	1.05	1.03 – 1.07	<0.001			
Anxiety/Depression^c^	0.90	0.83 – 0.97	0.009	0.87	0.77 – 0.99	0.036
Thinking disturbance^c^	1.21	1.11 – 1.32	<0.001	1.16	1.04 – 1.30	0.010
**Hostility/Suspicion§**^**c**^	1.29	1.17 – 1.42	<0.001	**1.29**	**1.14 – 1.45**	**<0.001**
Activation^c^	1.11	1.02 – 1.21	0.011	0.87	0.77 – 0.99	0.036
*NPI-29 scores*						
Total	1.13	1.06 – 1.21	<0.001			
Leadership (factor 1)^d^	1.28	1.09 – 1.51	0.003	1.28	1.09 – 1.51	0.003
Exhibitionism (factor 2)^d^	1.27	1.07 – 1.51	0.007	1.27	1.07 – 1.51	0.007
**Superiority (factors 3)**^**d**^	1.41	1.13 – 1.75	0.002	**1.41**	**1.13 – 1.75**	**0.002**
**Entitlement (factor 4)**^**d**^	1.36	1.14 – 1.63	0.001	**1.36**	**1.14 – 1.63**	**0.001**
				**Final multivariate analysis**		
**Not in paid work**				3.13	1.21 – 8.10	0.019
**Compulsory admission**				4.95	2.18 – 11.23	<0.001
GAF-Function				1.01	0.97 – 1.04	0.746
**BPRS Hostility**				1.20	1.07 – 1.34	0.001
NPI-29 Superiority				1.13	0.86 – 1.48	0.385
**NPI-29 Entitlement**				1.23	0.99 – 1.53	0.013

*For groups of variables as indicated: ^a^Education and work ^b^Admission and diagnosis ^c^BPRS subscales ^d^NPI-29 subscales # Fat types: Variables included in the final multivariate analysis. § Violence and BPRS Hostility/suspiciousness correlate rho = 0.60, and therefore only the latter variable entered the final multivariate analysis.

## Discussion

This study report that high perceived humiliation during the admission process was significantly associated both with socio-demographic variables like low education and not being in paid work, and with psychopathology such as a diagnosis of schizophrenia, compulsory admission, severe violence, lower mean GAF-Function and GAF-Symptom scores, lower total and subscales mean BPRS scores and higher mean NPI-29 total and subscale scores. In the final multivariate model not being in paid work, compulsory admission, BPRS Hostility/Suspiciousness and NPI-29 Entitlement/Uniqueness were significantly associated with high humiliation.

Most previous studies of the admission process to psychiatric institutions have focused on coercion or perceived coercion, which concerns the patients’ convictions that they do not have influence, control, freedom, or choice, as they do not themselves make the decision concerning admission [20]. While the perception of coercion is the cognitive appraisal of such admissions, humiliation can be seen as the appraisal’s corresponding emotional reaction. Like Rooske & Birchwood [4] we found that humiliation was strongly associated with compulsory admission and with a diagnosis of schizophrenia. We did not find any significant association between self-rated depression (HADS) and humiliation. Birchwood et al. [3,21] found that patients developing post psychosis depression reported experiencing humiliation prior to the depression. Their patients also were more likely to see themselves as having a “lower rank status”. This result supplements ours from the acute admission phase, and confirms our previous finding that humiliation was not reduced in the “less improved” patients [6]. One possible explanation, due to the pathogenic nature of humiliation, is that the depressive symptoms were initiated or maintained by the humiliating experience.

We observed that not being in paid work was strongly associated with high humiliation, and we reasoned that a psychiatric admission would be an additional blow to an individual with an already reduced self-esteem as an additional loss of social status. Our finding of a significant association between low education and humiliation is in accordance with Rooske & Birchwood [4] reporting that humiliation and lower rank status are associated with development of post psychosis depression. A compulsory versus voluntary admission was also strongly associated with high humiliation, which we have reported in a previous paper [6]. A compulsory admission is an experience that seems to hit the central core of the unjust perspective of humiliation [1] as well as the status loss perspective [2].

Our cross-sectional study cannot clarify the causality of humiliation, but relations may be bidirectional. The process of compulsory admission for mental disorders fulfils the descriptions of the humiliation process. On the other hand, during their previous life history many patients with mental disorders already are established in humiliated position since they already feel unjustly treated or have experienced status losses. Therefore in such patients it is important to explore ways to not add insult to injury [2].

Some of our findings are consistent with the perspectives of Torres & Bergner and Lazare [1,2]. A higher score on the NPI-29 subscales of Superiority/Arrogance (Factor 3) and Entitlement/Uniqueness (Factor 4) i.e. excessive status claiming, showed strong associations with high humiliation. Patients with a high score on the BPRS Hostility/Suspiciousness subscale showed a high humiliation score. Rage is an emotional reactions strongly associated with humiliation [1]. Rage based on humiliation sometimes is acted out as violence [22,23], and severe violence showed a high correlation (rho 0.60) with violence in our sample [6]. Accordingly, finding ways to reduce humiliation in the compulsory admission process may serve to reduce the risk of violent behaviour.

Norway has a relatively high rate of compulsory admission compared to countries with similarities in legislation, and the Government considers a reduction of that rate as an important health-political aim. We have focused on the handling of patients during the sensitive process of psychiatric admissions, especially compulsory ones. We have emphasized the high risk of patient’s humiliation during these procedures, and that police and health care professionals have to be conscious about this aspect.

Perhaps special precautions and eventual intensive work towards voluntary admission should be taken when the individual is not in paid work, hostile or suspicious or express entitlement or superiority. Reducing perceived humiliation could lead to a reduction in compulsory admissions, a reduction in violence during the admission process and better treatment compliance. Certain patients with certain profiles seem to be especially prone to experience humiliation as a result of compulsory admission. Given the high pathogenic potential of humiliation, the exploration of ways to eliminate, control or mitigate humiliation in these patients, should be of interest in future research.

Adshead [24] points to humiliation’s impact on identity which is another reason to reduce humiliating events in psychiatry.

### Strengths and limitations

Our findings must be considered in the light of several limitations. The exclusion of manic/hypomanic states and severe cognitive impairment could be considered as both strength and a limitation. It is strength because their lack of critique makes self-rating of symptoms and humiliations less meaningful. The exclusion is also a limitation since comparison of their reports compared to other diagnostic groups had been of considerable interest. The same arguments can be used from the exclusion of patients with confusion and cognitive impairment.

Another major limitation is the measurement of humiliation. We had no objective way to measure this perception and reports are based on the subject’s individual experience and understanding of humiliation. Although the patients were informed of a simple definition of humiliation: “humiliation is to feel that you are put down or made to feel inferior”, there is still doubts concerning what they are reporting. The first author has explored the concept of humiliation with patients and earlier psychiatric patients and they have confirmed the understanding of humiliation as “being put down” or “made to feel inferior” [5]. This confirmation is lacking further replication. We regard our choice as preliminary and in need of further exploration in order to find the most valid way of measuring humiliation. The instrument developed by Hartling [25] was not known to us when our study was designed.

Self-rating made by severe ill patients is an issue open to discussion, and concern the NPI-29 and the HADS in our study. The patients in the presence of psychiatric nurses rated these scales, so they could ask for help if needed. To the nurses it seemed that these scales were feasible for the patients. The criterion needed for valid ratings is that the patients intellectually understand the content of the questions. Ego-syntonic traits and motivations influence the self-rating of anyone, not just patients with severe mental disorders. A recent study by Lincoln et al. [26] tested the correlations of observer-based rating and patient self-ratings in regard to delusions. The correlations ranged from 0.49 to 0.57 (with explained variance of 24% to 39.5%), and they conclude that patients provide reliable information. We therefore consider that self-rating of symptoms by psychotic patients, excluding the diagnoses mentioned, has some value, and when they rate their level of humiliation we consider that random error rather than bias are operating.

We consider it a strength that outcome was measured both by interview-based (BPRS) and self-rated instruments (HADS) that are sensitive to change and with good psychometric properties. It can be questioned whether the patients report humiliation perceived from the admission process or the inpatient facilities. Since the patients were interviewed within 48 hours it is likely that some of the reports are from the admission process and some from the 48 hours in the hospital.

## Conclusion

In our sample of psychiatric patients evaluated during the admission process to acute hospital units, high perceived humiliation was associated with compulsory admission, not being in paid work, showing hostility or suspicion and expressing superiority or entitlement. Individuals in any of these categories may be particularly prone to experience humiliation. Compulsory admissions should, as far as possible, be avoided. Precautions should be taken in order to identify patients vulnerable for experiencing humiliation, and the factors we have identified may be of help in the clinic. Inpatient therapy should explore humiliation and help patients to understand the phenomenon in various perspectives. This may help individuals to restore aspects of their identity and cope with the strong feelings of humiliation that some patients experience.

## Competing interests

The authors declare that they have no competing interests.

## Authors’ contributions

MFS contributed to conception and design of the study, acquisition of data, statistical analysis and interpretation of data, as well as drafting and revising of the manuscript. JAN contributed to conception and design, and revised the manuscript critically. AAD contributed to conception and design, statistical analysis and interpretation of data, as well as drafting and revising the manuscript. All authors have approved the final version of the manuscript.

## Pre-publication history

The pre-publication history for this paper can be accessed here:

http://www.biomedcentral.com/1471-244X/13/217/prepub
